# Salivary signatures of oral-brain communication in sleep bruxers

**DOI:** 10.3389/fcimb.2023.1321855

**Published:** 2023-12-06

**Authors:** Yuwei Deng, Chenyuan Zhu, Ruixue Jiang, Chunhua Yu, Jun Zhao, Xinquan Jiang, Jin Wen

**Affiliations:** ^1^ Department of Prosthodontics, Shanghai Ninth People's Hospital, Shanghai Jiao Tong University School of Medicine, Shanghai, China; ^2^ College of Stomatology, Shanghai Jiao Tong University, National Center for Stomatology, National Clinical Research Center for Oral Diseases, Shanghai, China; ^3^ Shanghai Key Laboratory of Stomatology, Shanghai Research Institute of Stomatology, Shanghai Engineering Research Center of Advanced Dental Technology and Materials, Shanghai, China

**Keywords:** metagenomics, metabolomics, oral microbiome, GlcNAc, neuro-immune regulatory network

## Abstract

**Introduction:**

Microbiota and their interaction with hosts have been of great interest in brain research in recent years. However, the role of oral microbiota in mental illness and the underlying mechanism of oral-brain communication remains elusive. Sleep bruxism (SB) is an oral parafunctional activity related to the nervous system and is considered a risk factor for harmful clinical consequences and severe systemic conditions. Exploring the connection between oral microbiota and sleep bruxism may deepen our understanding of the complex relationship between oral-brain axis and provide insights for treatment.

**Methods:**

In this study, salivary samples were collected from 22 individuals with SB and 21 healthy controls, and metagenomics with metabolomics was performed. Nonparametric Wilcoxon test were applied for the statistical analysis between the two groups. Microbial dysbiosis and altered oral metabolites were found in the SB individuals.

**Results:**

The characteristic metabolite N-acetylglucosamine (GlcNAc) (VIP=8.4823, P<0.05) was correlated to a statistically lower Streptococcus mitis level in SB individuals. Salivary IFN-g level and IFN-g/IL-4 ratio were detected with significant changes in a chip assay. Amino acid metabolism pathways were upregulated, and the pathway with the largest number of differentially expressed genes is related to amino-tRNA charging pathway, while the most significantly enriched pathway is related to arginine biosynthesis. Neurotransmitter-associated pathways with glutamatergic and GABAergic synapses and cardiovascular system-related pathways were enriched in the SB group.

**Discussion:**

These results indicate a possible neuroimmune regulatory network of oral-brain communication in SB, which helps explain the mechanism of the oral microbiome with the host in sleep bruxers and provides a reference for early clinical and therapeutic intervention to improve the diagnosis and treatment of SB and similar diseases.

## Introduction

Abundant and diverse microbial communities coexist in the human body (Sorboni et al., 2022). Microbial community and host crosstalk have powerful functions related to human health status and the progression of systemic disorders (Deo and Deshmukh, 2019; Graves et al., 2019; Peng et al., 2022). Over the past two decades, accumulating reports have revealed that the gut microbiota and microbiome mediate several physiological processes, such as the maintenance of homeostasis, immunomodulation, and regulation of the central and enteric nervous systems. With these insights, the microbiota-gut-brain axis (MGBA) was proposed to illustrate the connection between dysbiosis of the gut microflora and mental health (Cryan et al., 2020; Maitre et al., 2020). Increasing clinical and preclinical evidence has indicated that the altered composition of gut microbiome contributed to the pathophysiology of neuropsychiatric disorders (NPDs), such as alzheimer’s disease (AD), multiple sclerosis, and parkinson’s disease (Nair et al., 2018; Järbrink-Sehgal and Andreasson, 2020). Additionally, mental status, such as stress, anxiety, and depression, have been reported as critical susceptibility factors for some gut diseases (e.g., irritable bowel syndrome and ulcerative colitis) (Sorboni et al., 2022).

Similarly, as the second largest and most diverse microbiota in the human body, the oral microbiota and its derived metabolites interact closely with hosts (Peng et al., 2022). Despite the consensus that oral bacteria are closely linked to oral diseases (Giordano-Kelhoffer et al., 2022), new evidence has been reported suggesting that mental health and oral diseases are closely interconnected due to shared social determinants (Peng et al., 2022; Joury et al., 2023). Anxiety and depressive-like behaviors have been observed in periodontitis animal models (Martínez et al., 2021). Epidemiological studies have confirmed an association between periodontitis and NPDs (Bulgart et al., 2020). Furthermore, the oral microbe *P. gingivalis* has been known to directly affect AD progression (Dominy et al., 2019). An increasing amount of research has provided support for the existence of oral-brain communication (Simpson et al., 2020; Abdalla et al., 2022).

As a repetitive masticatory muscle activity during sleep, sleep bruxism (SB) is an oral parafunctional activity related to the nervous system (Polmann et al., 2021). It is considered as a risk factor for harmful clinical consequences, including abnormal wear of the teeth, disorders of the temporomandibular system, and pain in the masticatory muscles (Lobbezoo et al., 2018), and causes a heavy burden for patients. A multifactorial etiology has been widely reported, including biological, psychological, and exogenous factors (Bertazzo-Silveira et al., 2017). Increasing evidence supports a positive association between SB and psychological symptoms (Polmann et al., 2019; Polmann et al., 2021). Although some theories have been proposed to explain the interactions between the oral cavity and the central nervous system (Bowland and Weyrich, 2022), clinical and preclinical studies are limited compared with studies on the MGBA. Therefore, little is known and evidence is limited about the underlying mechanism of the oral-brain axis in sleep bruxers.

In this study, we performed an integrated analysis of salivary metagenomics and metabolomics to compare the salivary signatures in SB individuals compared with healthy individuals to explore the role of microbial and host metabolites in SB individuals. Combined with the detection of the salivary cytokines and in-depth Ingenuity Pathway Analysis (IPA), this study aimed to explore the underlying mechanism of oral-brain communication in sleep bruxers and provides a reference for novel intervention strategies to improve SB and related disease outcomes.

## Materials and methods

Human ethics approval for this study was granted by the Ethics Committee of the Ninth people’s hospital affiliated with the Shanghai Jiao Tong University School of Medicine (Approval number: SH9H-2022-T280-1). All participants were informed of the procedure of this experiment and voluntarily signed informed consent. Inclusion criteria: Patients who met the inclusion criteria (**Table 1**) were eligible for the recruitment. The diagnostic criteria accorded with the American Academy of Sleep Medicine(AASM) report (Manfredini et al., 2017).

**Table 1:** The inclusion/exclusion criteria in the current study.

Inclusion criteria		Exclusion criteria
The Bru group	The Nor group	
1) Diagnosed as SB within the past six months, including phasic, tonic, and mixed bruxism. 2) Abnormal wear of teeth is consistent with at least one of the following conditions: ①Horizontal teeth wear corresponded to the trajectory of forward and lateral movement; ② Excessive cup-shaped wear on the occlusal surface. 3) Existing one of the following symptoms &signs: ①Morning discomfort of maxillofacial muscles; ② Hypertrophy of masseter and/or temporalis muscle; ③ Alveolar bone hyperplasia; 4) Healthy periodontal and mucosal tissue; 5) Aged 18-40 years.	1) Complete dentition without teeth wear; 2) Healthy periodontal and mucosal tissue; 3) Aged 18-40 years.	1) System diseases; 2) Teeth wear combined with acid erosion; 3) Existing one of the following conditions: remaining untreated caries; received periodontal treatment within two months; confirmed temporomandibular joint disorder (TMD); history of orthodontic treatment; wearing fixed or removable restoration; 4) Use of antibiotics or hormone drugs within the past six months; 5) Pregnant women; 6) Smokers.

### Salivary collection

The collection of each saliva sample followed the following requirements: (1) This work was scheduled between 09:00 and 11:00 a.m. (2) Each participant was restrained from food and water for at least 1 hour before salivary collection. (3) Before collection, each participant needed to clean his/her hands with an alcoholic disinfectant. (4) After rinsing their mouth with deionized water for 1 min, the participants were instructed to spit the saliva into a sterilized centrifuge tube until 6-8 mL saliva was collected. Make sure to measure fluid level rather than foam. Most people take 2 to 5 minutes to fill the tube. (5) Once enough saliva have collected, screw the cap back onto the tube tightly. Each saliva sample was immediately stored in aliquots at −80 °C until further investigation.

### Metagenomic sequencing

The sequencing libraries were acquired by NEBNext® UltraTM DNA Library Prep Kit for Illumina (NEB, USA) following the manufacturer’s recommendations. Raw Data was obtained from the Illumina HiSeq sequencing platform, and then Clean Data was obtained by applying the Readfq (V8, https://github.com/cjfields/readfq) to preprocess the Raw Data. The Scaftigs (> 500 bp) that were generated from the both single and mixed assembly were all performed subsequent gene prediction by MetaGeneMark (V2.10, http://topaz.gatech.edu/GeneMark/) software. For open reading frame (ORF) prediction, the Scaftigs were adopted to de-redundancy by CD-HIT software (V4.5.8, http://www.bioinformatics.org/cd-hit) and constructed the unique initial gene catalog. Clean Data of each sample was mapped to the initial gene catalog by Bowtie2 (Bowtie2.2.4) and compared with the MicroNR database.

### Sample preparation and metabolomic analysis

The aliquot of salivary samples was thawed and centrifuged at 1500 g for 15 min at 4 °C. 100 μL supernatant was vortex mixed with pre-cooled methanol/acetonitrile/water solution (2:2:1, v/v), oscillated, low temperature ultrasound-treated for 30 minutes, stood for 10 min at -20°C, 14000 g centrifuged at 4°C for 20 min. The supernatant was collected for untargeted ultra-performance liquid chromatography quadrupole time-of-flight mass spectrometry (UPLC-QTOF/MS) analysis.

The samples were separated by a UHPLC (1290 Infinity LC, Agilent Technologies). The Mass spectrometry was performed by the coupled quadrupole time-of-flight (AB Sciex TripleTOF 6600) in Shanghai Applied Protein Technology Co., Ltd. QC samples were inserted into the sample queue to monitor and evaluate the stability of the system and the reliability of the experimental data. The resulting data was imported into freely available XCMS software for data analysis.

### Luminex liquid suspension chip detection

To measure cytokine levels in saliva, Luminex liquid suspension chip detection was performed by Wayen Biotechnologies (Shanghai, China). The Bio-Plex Pro Human Cytokine Grp I Panel 27-plex was applied according to the manufacturer’s protocol. The salivary samples were centrifuged at 10,000 rpm for 10 min, and the supernatant was collected. 50 µL of each sample was added to a 96-well plate embedded with microbeads and incubated for 30 min. Next, 25 µL of diluted detection antibody was added and incubated for 30 min. After incubating with Streptavidin-PE for 10 min, the fluorescence value was detected using the Bio-Plex MAGPIX System (Bio-Rad). Cytokines including IFN-γ, IL-4, IL-6, IL-8, IL-17 and TNF-α were detected.

### Bioinformatic analysis and statistical analysis

Nonparametric Wilcoxon test and Anosim analysis (R vegan package, Version 2.15.3) were applied for the statistical analysis between the two groups. Metastats analysis was performed for each taxonomy by Permutation test. The p-value was corrected by Benjamini and Hochberg False Discovery Rate, and then the q value was acquired for screening the different species between groups. Variable importance of projection (VIP) values obtained from the orthogonal partial least-squares discriminant analysis (OPLS-DA) model was used to screen significantly changed metabolites (VIP > 1, p-value < 0.05). Spearman correlation analysis was used to calculate the correlation coefficient between the variables of microbiome and metabolites. Functional annotation and abundance analysis of KEGG (Kyoto Encyclopedia of Genes and Genomes, http://www.kegg.jp/) pathways were carried out based on the data. IPA (QIAGEN) was applied to identify the significant top canonical pathways and disease/function annotation with data of differentially expressed metabolites.

## Results

### Taxonomy annotation of the microbial community structure

**Figure 1:**
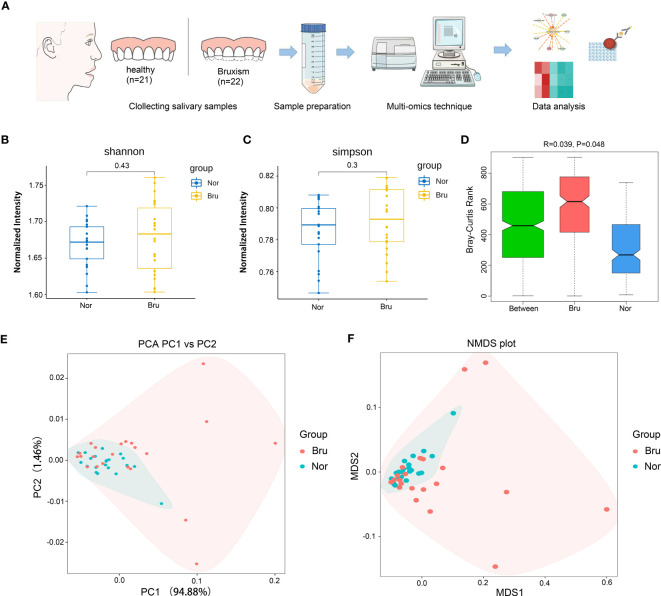
Basic information and diversity of salivary microbiota. **(A)** The Flow chart clarified the process of this study. **(B, C)** Shannon index and Simpson index show the a diversity. **(D)** Anosim analysis results (R=0.039, P=0.048) indicated significant differences between groups. **(E)** The PCA plot of the two groups according to the abundance table of taxonomic hierarchy at the species level. **(F)** The NMDS plot of the two groups at the species level. (Nor, normal group; Bru, Sleep bruxism group).

Forty-three participants who met the inclusion and exclusion criteria in **Table 1** were recruited in this study, including 22 sleep bruxers (Bru Group) and 21 healthy controls (Nor group). The process for this study is shown in **Figure 1A**. A total of 4 kindom, 130 phyla, 107 classes, 246 orders, 552 families, 1887 genera, and 7981 species were detected. The Shannon index and Simpson index were used to illustrate the α diversity of the microbial community richness. There were no significant differences between the two groups (P>0.05) (**Figures 1B, C**). Anosim analysis results (R=0.039, P=0.048) indicated significant differences between groups (**Figure 1D**) and a statistically significant separation of the two groups. The more similar the species composition of the samples, the closer the distance between each sample, as shown in the principal component analysis (PCA) and non-metric multidimensional scaling plots (**Figures 1E, F**). The results suggested a divergent trend between the two groups.

**Figure 2:**
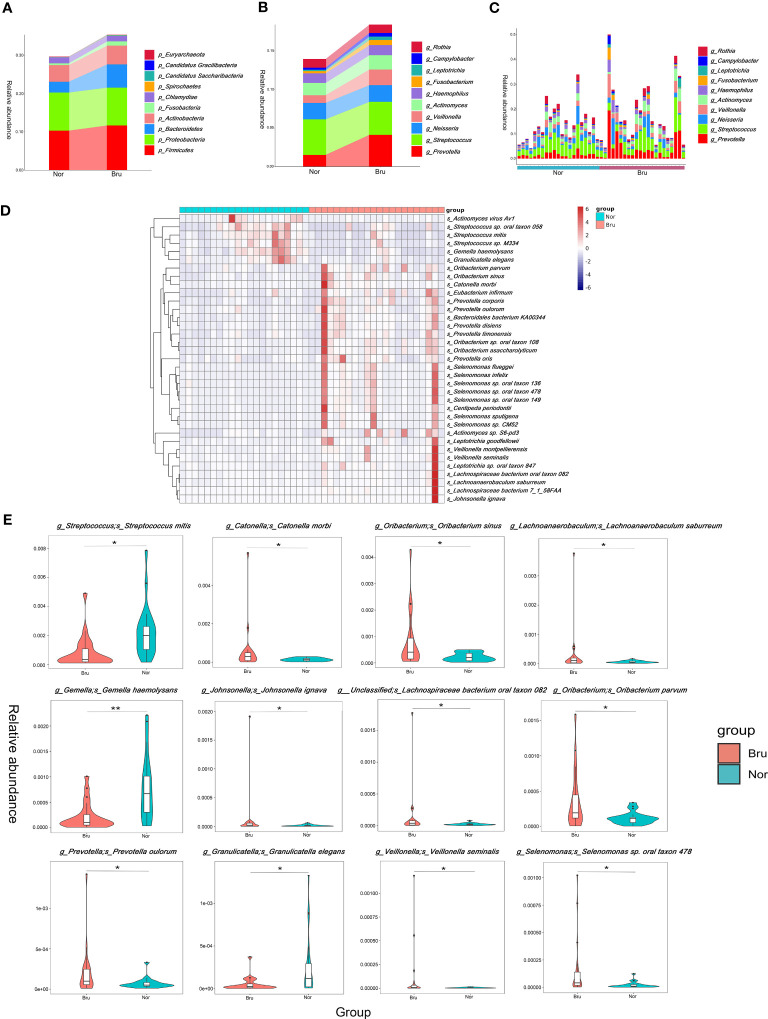
The data analysis of Metagenomics. **(A)** The top ten abundant phyla in the two groups. **(B)** The dominant ten genera in the two groups. **(C)** The relative abundance of each sample at the genera level. **(D)** The top 35 abundance cluster heat map according to Metastats analysis. The corresponding value of the heat map presents the Z value of the relative abundance of species in each row after standardized processing. **(E)** The box diagram of the Top12 significantly different species. The vertical axis presented the relative abundance of the corresponding species. (* q value<0.05; ** q value<0.01).

Based on the relative abundances, the top 10 abundant phyla in the two groups were *Firmicutes, Proteobacteria, Bacteroidetes, Actinobacteria, Fusobacteria, Chlamydiae, Spirochaetes, Candidatus Saccharibacteria, Candidatus Gracilibacteria, Euryarchaeota* (**Figure 2A**). The results showed increased richness within the Bru group of the *Firmicutes*, *Bacteroidetes*, and *Fusobacteria*. The top 10 abundant genera and the relative abundance at the genera level are listed in **Figures 2B, C**.

To compare the differences in absolute abundance between the two groups and identify species with substantial differences, Metastats analysis was performed at the different levels. A total of 11 phyla, 140 genera, and 1233 species revealed different abundances. Abundance cluster heat map analysis at the species level are shown in **Figure 2D**. At the phylum level, the relative abundances of *Bacteroidetes, Fibrobacteres*, and *Synergistetes* in the Bru group were significantly higher than in the Nor group. At the species level, the relative abundance of *Streptococcus mitis* in the Bru group was significantly lower than in the Nor group. The relative abundances of *Catonella morbi* and *Oribacterium sinus* in the Bru group were considerably higher than in the Nor group. The box diagram of the top 12 significantly different species is shown in **Figure 2E**. These data together suggested a state of microbial dysbiosis in the microbiome community of sleep bruxers.

### Characterization of metabolites in saliva samples

**Figure 3:**
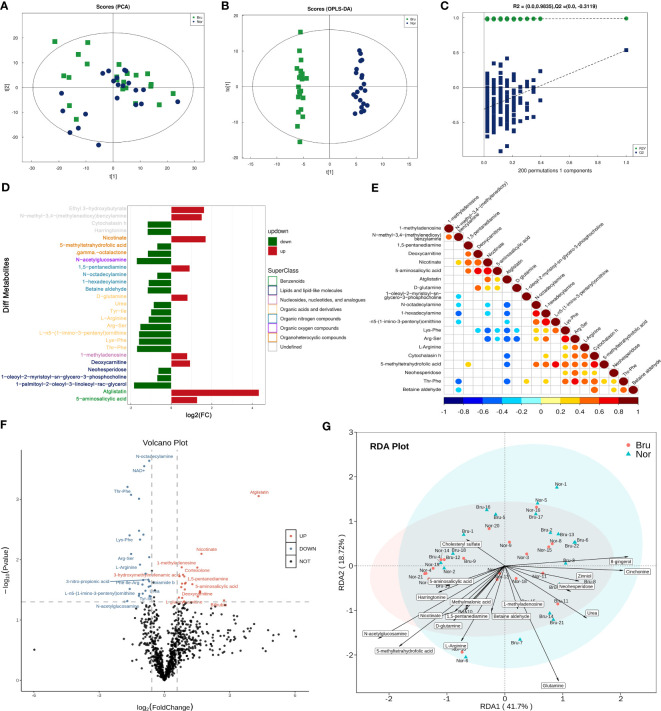
The data analysis of Metabolomics. **(A)** The PCA Scores of the metabolites in the two groups. **(B)** The OPLS-DA Scores of the metabolites in the two groups. (R2Y = 0.991, Q2 = 0.536) **(C)** The Permutation test diagram of the OPLS-DA model revealed no overfitting and a reliable test result. +**(D)** A bar chart demonstrates the changes in the multiple significant metabolic differences. **(E)** Correlation analysis of differential metabolites. Red indicates a positive correlation, blue indicates a negative correlation, and white indicates a non-significant correlation. **(F)** The volcano map showed those upregulated(Red) and down-regulated(Blue) differential metabolites based on the univariate Statistical Analysis. **(G)** The differential metabolites were taken as environmental factors, and RDA analysis was performed at the species level.

The PCA model parameters obtained through a 7-fold cross-validation are shown in **Figure 3A**. OPLS-DA were also carried out to demonstrate the overall sample distribution (**Figure 3B**). The apparent separation between groups indicated that the model was reliable (R2Y = 0.991, Q2 = 0.536) . The Permutation test diagram of the OPLS-DA model also revealed no overfitting and a reliable test result. (**Figure 3C**). This indicated that the state of the microbial environment in SB individuals changed the abundance of metabolites. A bar chart was used to visually demonstrate the changes in multiple significant metabolic differences (**Figure 3D**). According to the VIP value, the top differential metabolites included deoxycarnitine, N-acetylglucosamine (GlcNAc), L-arginine, and Thr-Phe. (**Supplementary Table 1**). Correlation analysis of differential metabolites clarified the regulatory relationships between metabolites during the biological process. The results of the correlation analysis were visualized in the form of a correlation heat map (**Figure 3E**). The volcano map shows the upregulated (red) and downregulated (blue) differential metabolites in **Figure 3F**. Redundancy analysis was performed at the species level by taking those differential metabolites as environmental factors (**Figure 3G**).

### Pathways and enrichment

**Figure 4:**
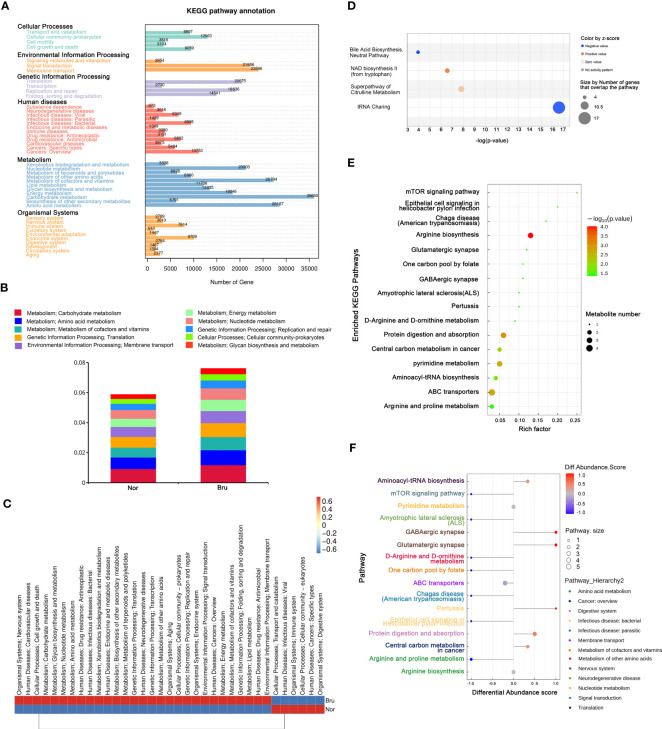
The enriched pathway involved in SB. **(A)** The KEGG pathway annotation based on the results of Unigenes annotation. The numbers on the bar chart represent the number of Unigenes on the annotation. **(B)** The top 10 predicted pathways for microbial function in the KEGG database between the two groups. **(C)** The heatmap of the KEGG pathways between the two groups. **(D)** Regarding top canonical pathways, IPA analysis revealed that the significantly deactivated pathway was tRNA Charging. **(E)** KEGG Enrichment pathways that relate to metabolism processing. The bubble map was illustrated based on the P value and enrichment factor. The color of the bubble represents the p-value of enrichment analysis. The color transitions from green to red; red indicates a smaller P-value and a more significant enrichment degree. **(F)** Differential abundance score map of enriched metabolic pathways.

Based on the results of unigene annotations, the predicted pathways for microbial function in the KEGG database are illustrated in **Figure 4A**, including carbohydrate metabolism and amino acid metabolism. Both the carbohydrate metabolism and amino acid metabolism pathways were remarkably differentiated and had the most significant number of expressed genes, which were upregulated in sleep bruxers (**Figures 4A, B**). The heatmap of the KEGG pathways between the two groups is shown in **Figure 4C**. The top canonical pathways of IPA analysis revealed that the most significantly deactivated pathway was tRNA charging (**Figure 4D**). The KEGG pathways related to the metabolism processing were illustrated by a bubble plot of metabolite set enrichment analysis (**Figure 4E**) and a differential abundance score map of enriched metabolic pathways (**Figure 4F**). Sixteen metabolic pathways were involved in SB. Among them, arginine biosynthesis was the most significantly differentially enriched metabolic pathway. Furthermore, arginine and proline metabolism pathways were significantly downregulated, and glutamatergic synapse and GABAergic synapse pathways were significantly upregulated. According to their upper pathway hierarchy classification, these last two pathways involved amino acid metabolism and the nervous system, respectively. This result was consistent with the KEGG annotations and confirmed communication through the oral-brain axis.

### Correlation between microbiomes and metabolomics

**Figure 5:**
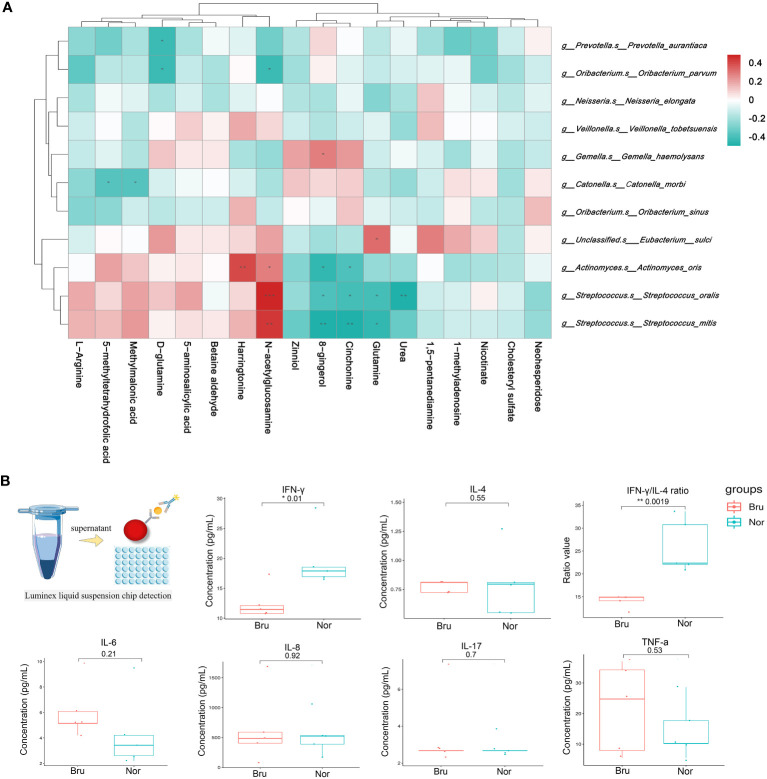
Correlation analysis of microbiomes and metabolomics. **(A)** Correlation between microbiomes and metabolomics. The color of the heatmap indicates the correlation coefficient R. R>0 means a positive correlation, shown in red; R<0 represents a negative correlation, which is shown in blue. **(B)** The concentration of salivary cytokines which detected by Luminex liquid suspension chip detection. (* P value<0.05; ** P value<0.01).

The spearman analysis method was used to calculate the correlation coefficient between the variables of microbiome and metabolites. The result was visualized in the form of a cluster heat map (**Figure 5A**). At the species level, *Streptococcus mitis, Streptococcus oralis, and Actinomyces oris* had the closest correlations with differential metabolites, including GlcNAc, glutamine, and urea.

### The concentration of salivary cytokines

Luminex liquid suspension chip detection was used to measure the concentration of salivary cytokines (**Figure 5B**). The experimental results were repeated and met quality control requirements. The results showed that the proinflammatory factor IFN-γ (Nor: 19.67 ± 4.94 pg/mL; Bru: 12.48 ± 2.72 pg/mL, P= 0.01) significantly decreased in the Bru group, while there was no significant difference in proinflammatory cytokines such as IL-4, IL-8, IL-17, and TNF-α between the two groups. The IFN-γ/IL-4 ratio (Nor: 25.97 ± 5.84; Bru: 14.12 ± 1.42, P= 0.0019) significantly decreased in the Bru group. These data together confirmed changes in salivary inflammatory cytokines in sleep bruxers.

### IPA analysis

**Figure 6:**
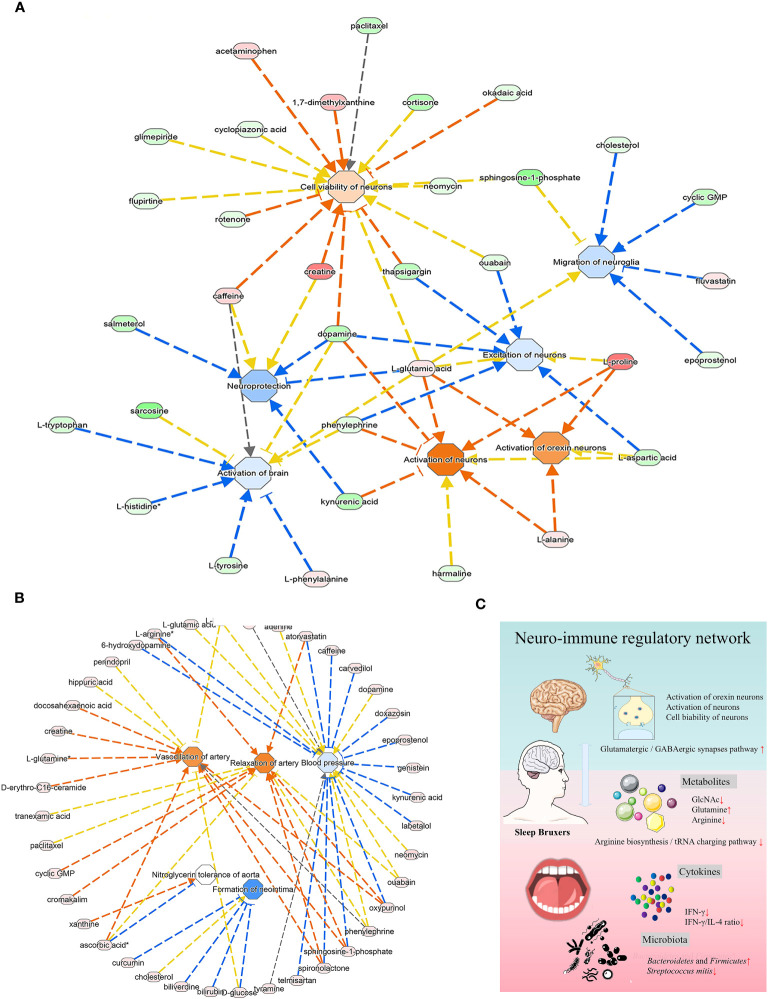
The analysis of underlying association of SB with the cardiovascular system. **(A)** The relationship between activation and inhibition of metabolites and function of the neurological system. **(B)** The predicted physiological system showed significant enrichment in the cardiovascular system. **(A, B)** The orange line signifies that changes in gene expression levels activate this function, while the blue line indicates that changes in gene expression levels inhibit this function. The yellow line represents an inconsistent impact of changes in gene expression levels on this function compared to existing literature reports, and the gray line denotes unknown regulatory relationships. **(C)** Schematic illustration briefly explained the underlying mechanism of oral-brain communication involved in sleep bruxism.

The predicted diseases and disorders were associated with immunological, inflammatory, and neurological conditions. The activated pathways related to the neurological system included activation of the brain, excitation of neurons, neuroprotection, and migration of neuroglia (**Figure 6A**). The IPA analysis predicted a suggestive association between SB and the cardiovascular system, in which the significantly upregulated pathway was vasodilation of the artery and relaxation of the artery. In contrast, blood pressure and neointima formation were predicted to be substantially downregulated (**Figure 6B**).

## Discussion

With the development of new techniques, the crosstalk between the oral cavity and the host and the possible effects of biochemical activities in sleep bruxers can be further explored by integrated analysis of metagenomics and metabolomics. In this study, the β diversity reflecting the microorganism structure was significantly different between the two groups, and the relative abundance of *Bacteroidetes* at the phylum level in the Bru group was significantly higher than that in the Nor group, suggesting a state of microbial dysbiosis in the microbiome community of individuals with SB. Moreover, the imbalance of the microbial community not only related to differences in the abundance of functional genes in the oral environment, but also changed the metabolic capacity and led to differences in the abundance of metabolites. The PCA and OPLS-DA analysis (**Figures 3A, B**) of saliva metabolites demonstrated differentially expressed metabolites between SB individuals and controls, suggesting that apart from the state of microbial dysbiosis, altered oral metabolites were present in the SB individuals.

According to the enriched KEGG pathway annotation (**Figure 4A**), the carbohydrate and amino acid metabolism pathways were enriched with the most significant number of differentially expressed genes and were upregulated in sleep bruxers (**Figure 4C**), while protein digestion and adsorption were upregulated in Bru group, as shown in **Figures 4E, F**. Based on previous studies, one explanation is that the metabolism of these carbohydrates and amino acids provided carbon, nitrogen, and energy sources for bacteria or pathogens, maintaining the stability and function of the oral ecosystems. The availability of free amino acids is reported to play an essential role in resistance to environmental stresses (Christgen and Becker, 2019), which may explain the upregulated pathways in SB.

The most significant pathway related to metabolism processing was the arginine biosynthesis pathway, which was downregulated in the Bru groups (**Figures 4E, F**). Arginine was shown to be an essential substrate for regulating the balance of the oral microbiome and the dynamic balance of ions. The host cells also metabolize arginine by the arginase pathway to produce ornithine, urea, and NO (Carpenter, 2020). In accordance with the experimental results, decreased levels of ornithine and urea were detected in the Bru group (**Figures 3D, F**). As shown in **Figure 5A**, the correlation analysis showed that *Streptococcus mitis, Streptococcus oralis, and Actinomyces oris* had the most significant correlations with differentially expressed metabolites, including GlcNAc, glutamine, and urea, which are involved in SB. In our results, GlcNAc was significantly downregulated in the sleep bruxers and closely related to a statistically lower level of *Streptococcus mitis* (**Figure 2E**), which further confirms this correlation analysis.

In the human body, GlcN and GlcNAc are precursors of glycosaminoglycan synthesis, such as hyaluronic acid, chondroitin sulfate, and keratin sulfate, which maintain cartilage health and safeguard joint function (Yumoto et al., 2019). It was reported that GlcNAc displayed significant immunomodulatory activity by independently inhibiting proinflammatory type 1 and type 17 helper T cell responses to proinflammatory innate B cell activity(Grigorian et al., 2011). Sy et al. also reported GlcNA reduced pro-inflammatory responses, promoted myelin repair, and reduced neurodegeneration by crossing the blood-brain barrier directly (Sy et al., 2020). Moreover, GlcNAc deficiency has been reported to be associated with neurodegeneration in patients with demyelination (Brandt et al., 2021). This might explain why differentially expressed genes were found to be enriched in the immune system pathway (**Figure 4A**) and might be a direct communication of the oral-brain axis. The KEGG analysis and the results shown in **Figure 4D** also revealed that the differentially enriched metabolites were significantly associated with the aminoacyl tRNA biosynthesis and tRNA charging pathways, which function in the utilization and immunoregulation of amino acids, further confirming this potential immune connection. In the traditional opinion, SB was not described as involving chronic inflammation or even the immune system (Kelly and Pearce, 2020). By detecting uncharged tRNAs, general control nonderepressible 2 (GCN2) senses amino acid starvation, and mTORC1 further licenses immune cell activation only when amino acid resources are available (Saxton and Sabatini, 2017).

Inflammatory messenger cytokines are an essential part of the neuro-immune regulatory network in the body. More related evidence about this in SB has emerged recently, when researchers found that the concentrations of the inflammatory markers in urine samples, including 17-hydroxycorticosteroids, C-reactive protein, and fibrinogen, were positively associated with the bruxism episode index. The salivary cortisol levels were also higher in subjects with SB (Flueraşu et al., 2021). To further confirm this immune connection in our study, we tested the salivary inflammatory mediators in the Bru group. Luminex liquid suspension chip detection was conducted, and alterations of stress-related cytokines (Takemori et al., 2021) like IFN-γ, IL-4, and IL-6 confirmed changes in the inflammatory environment in oral salivary. The anti-inflammatory factor IL-6 increased in the Bru group, IFN-γ (Nor: 19.67 ± 4.94 pg/mL; Bru: 12.48 ± 2.72 pg/mL, P= 0.01) and the IFN-γ/IL-4 ratio (Nor: 25.97 ± 5.84; Bru: 14.12 ± 1.42, P= 0.0019) significantly decreased in the Bru group. The IFN-γ/IL-4 ratio, as a potential biomarker, reflected the imbalance of Th1 and Th2 cytokine in the saliva, and was also reported to be negatively correlated with psychological stress (Chrousos, 2000; Takemori et al., 2021), This is explained by the downregulated arginine biosynthesis in the Bru group, which further inhibited the production of IFN-γ cytokine (Oberlies et al., 2009). IFN-γ might affect SB by regulating the serotonin (5-HT) receptor expression, as the serotonergic pathway was positively correlated with SB severity in individuals (Minakuchi et al., 2014).

Through the KEGG and IPA analysis, we also noted a potential interaction of the oral microbiome with the nervous system via a neurotransmitter-associated pathway. The enriched KEGG pathway in the current study was associated with glutamatergic and GABAergic synapses (**Figures 4E, F**). Glutamic acid and gamma-aminobutyric acid (GABA) are important excitatory and inhibitory neurotransmitters in the nervous system, respectively, and are essential for maintaining a synaptic transmission excitatory and inhibitory balance (O'Mahony et al., 2015). These results were similar to the report that sleep bruxism was related to disturbances in GABAergic and glutamatergic systems (Dharmadhikari et al., 2015). The enriched *Bacteroidetes* and *Firmicutes* could produce additional GABA, which was reported to promote sleep activity in the brain, and was thought to play a vital role in bruxism (Manfredini and Lobbezoo, 2009). Screening the related KEGG maps (hsa04724, hsa04727) suggested a regulation in glial cells and neurons, and the increased glutamine participated in this process. IPA analysis showed consistent results (**Figure 6A**), suggesting brain activation and excitation of neurons. This enrichment of neurotransmitter-associated pathways confirmed the interaction of the oral microbiome with the brain through the nervous system, suggesting that the network of oral microbiota and metabolites in sleep bruxers might play a role in modulating the nervous system and brain function.

In addition, IPA analysis, as shown in **Figure 6A**, also presented a suggestive association of SB with the cardiovascular system, in which the artery vasodilation and relaxation pathway was significantly upregulated. In contrast, blood pressure and neointima formation were predicted to be substantially downregulated. Sleep bruxism is also reported to co-occur with other severe conditions, such as cardiovascular diseases (Michalek-Zrabkowska et al., 2021), digestive system disorders, and sleep apnea (Martynowicz et al., 2019; Kuang et al., 2022). This may be due to increased sympathetic nerve activity in bruxers that led to processes such as oxidative stress, endothelial remodeling, and hormonal disturbances (Michalek-Zrabkowska et al., 2021). More light should be shed on this connection in future studies. In our study, there were many limitations to providing more valuable information for bruxers, as this study lacked certain critical parameters. In the future, large-scale clinical cohort study can be carried out to monitor the sympathetic nerve activity of sleep bruxism patients by non-invasive methods like heart rate variability analysis, microneurography, and sympathetic nerve recordings, collect information such as lifestyle habits, mental status and immune disorders, and longitudinally track the development of cardiovascular diseases, digestive system disorders and sleep apnea, so as to unravel the intricate mechanisms underlying the relationship between sleep bruxism and the other diseases.

Thus, we presumed that a state of microbial dysbiosis and altered oral metabolites would cause a signature of low-grade inflammation in SB individuals. Combined with robust bioinformatics analysis, we concluded that the underlying mechanism of oral-brain communication in SB may be mainly related to GlcNAc and IFN-γ mediated immune network with the arginine biosynthesis and tRNA charging pathways. Neurotransmitter-associated pathways with synapses and the cardiovascular system may be related to SB crosstalk within the entire body (**Figure 6C**). Together, this study provided essential insights for studying salivary signatures and the possible neuro-immune regulatory network in SB, highlighting its capacity for early clinical and therapeutic intervention for related systemic disorders.

## Data availability statement

The data presented in the study are deposited in the National Center for Biotechnology Information repository, accession number PRJNA932961.

## Ethics statement

The studies involving humans were approved by the Ethics Committee of the Ninth People’s Hospital Affiliated with the Shanghai Jiao Tong University School of Medicine. The studies were conducted in accordance with the local legislation and institutional requirements. The participants provided their written informed consent to participate in this study. Written informed consent was obtained from the individual(s) for the publication of any potentially identifiable images or data included in this article.

## Author contributions

YD: Data curation, Investigation, Methodology, Software, Validation, Visualization, Writing – original draft. CZ: Data curation, Investigation, Methodology, Software, Validation, Visualization, Writing – original draft. RJ: Data curation, Investigation, Validation, Writing – original draft. CY: Data curation, Investigation, Validation, Writing – original draft. JZ: Data curation, Investigation, Validation, Writing – original draft, Methodology. XJ: Funding acquisition, Project administration, Resources, Supervision, Writing – review & editing. JW: Data curation, Funding acquisition, Methodology, Project administration, Resources, Supervision, Validation, Visualization, Conceptualization, Investigation, Writing – original draft, Writing – review & editing.
