# Supplementary material for: Salivary signatures of oral-brain communication in sleep bruxers
Source: Front Cell Infect Microbiol. 2023 Dec 6;13:1321855. doi: 10.3389/fcimb.2023.1321855 (PMC10731308; doi:10.3389/fcimb.2023.1321855)
Supplement: Supplementary file 1 [file Table_1.docx]

**Supplementary Table 1** The top differential metabolites according to the VIP value.

| ID | Name | VIP | Fold change | p-value |
| --- | --- | --- | --- | --- |
| M146T369_2 | Deoxycarnitine | 10.56469744 | 1.910517015 | 0.031157457 |
| M465T245 | N-acetylglucosamine | 8.482321204 | 0.306254166 | 0.048557633 |
| M175T575 | L-Arginine | 7.571806953 | 0.459322286 | 0.012085625 |
| M267T208 | Thr-Phe | 6.056743723 | 0.308114965 | 0.000622404 |
| M567T25 | Atglistatin | 5.070847264 | 20.07486194 | 0.000884164 |
| M124T198 | Nicotinate | 3.526866239 | 3.249264795 | 0.008004817 |
| M61T97 | Urea | 3.194379404 | 0.545600858 | 0.032723982 |
| M103T296 | Betaine aldehyde | 2.892255682 | 0.445370468 | 0.003319635 |
| M262T431 | Arg-Ser | 2.81393873 | 0.345997991 | 0.008043495 |
| M295T186 | Tyr-Ile | 2.645254172 | 0.499701195 | 0.036565039 |
| M516T542 | Cytochalasin h | 2.374822476 | 0.445041198 | 0.000978487 |
| M602T31 | 1-palmitoyl-2-oleoyl-3-linoleoyl-rac-glycerol | 2.371010242 | 0.278512718 | 0.049757604 |
| M133T74 | Ethyl 3-hydroxybutyrate | 2.368853249 | 3.082574763 | 0.035129682 |
| M147T391 | D-glutamine | 1.97213409 | 1.750201561 | 0.02493465 |
| M349T393 | Neohesperidose | 1.94474296 | 0.639571499 | 0.027418488 |
| M86T254 | 1,5-pentanediamine | 1.905732288 | 1.883035361 | 0.018956432 |
| M270T131 | N-octadecylamine | 1.859769437 | 0.616242781 | 0.000229978 |
| M242T135 | 1-hexadecylamine | 1.667564617 | 0.4595712 | 0.00464901 |
| M282T287 | 1-methyladenosine | 1.633558921 | 1.731569871 | 0.013361314 |
| M460T436 | 5-methyltetrahydrofolic acid | 1.578971663 | 0.616536123 | 0.022940621 |
| M214T140 | L-n5-(1-imino-3-pentenyl)ornithine | 1.542038637 | 0.330745707 | 0.030581005 |
| M107T70 | .gamma.-octalactone | 1.528867883 | 0.447315759 | 0.036789554 |
| M733T98 | 1-oleoyl-2-myristoyl-sn-glycero-3-phosphocholine | 1.335298786 | 0.61608951 | 0.009098995 |
| M294T351 | Lys-Phe | 1.329117221 | 0.329380235 | 0.003954499 |
| M154T223 | 5-aminosalicylic acid | 1.167194475 | 2.431725148 | 0.025533653 |
| M166T154 | N-methyl-3,4-(methylenedioxy)benzylamine | 1.024900204 | 2.849945767 | 0.013530013 |
| M532T438 | Harringtonine | 1.016847027 | 0.44342346 | 0.039643559 |
